# *Galleria mellonella*: An Infection Model for Screening Compounds Against the *Mycobacterium tuberculosis* Complex

**DOI:** 10.3389/fmicb.2019.02630

**Published:** 2019-11-20

**Authors:** Masanori Asai, Yanwen Li, Jasmeet Singh Khara, Brian D. Robertson, Paul R. Langford, Sandra M. Newton

**Affiliations:** ^1^Section of Paediatric Infectious Diseases, Department of Infectious Disease, Imperial College London, London, United Kingdom; ^2^Department of Pharmacy, National University of Singapore, Singapore, Singapore; ^3^MRC Centre for Molecular Bacteriology and Infection, Department of Infectious Disease, Imperial College London, London, United Kingdom

**Keywords:** *Galleria mellonella*, tuberculosis, infection model, *Mycobacterium tuberculosis* complex, drug screening, antimycobacterial agents, mycobacteria

## Abstract

Drug screening models have a vital role in the development of novel antimycobacterial agents which are urgently needed to tackle drug-resistant tuberculosis (TB). We recently established the larvae of the insect *Galleria mellonella* (greater wax moth) as a novel infection model for the *Mycobacterium tuberculosis* complex. Here we demonstrate its use as a rapid and reproducible screen to evaluate antimycobacterial drug efficacy using larvae infected with bioluminescent *Mycobacterium bovis* BCG *lux*. Treatment improved larval survival outcome and, with the exception of pyrazinamide, was associated with a significant reduction in *in vivo* mycobacterial bioluminescence over a 96 h period compared to the untreated controls. Isoniazid and rifampicin displayed the greatest *in vivo* efficacy and survival outcome. Thus *G. mellonella,* infected with bioluminescent mycobacteria, can rapidly determine *in vivo* drug efficacy, and has the potential to significantly reduce and/or replace the number of animals used in TB research.

## Introduction

In 2017 alone, there were 1.6 million deaths from tuberculosis (TB), the leading global cause of infectious disease mortality (World Health Organization, 2018). It is estimated that approximately a quarter of the world’s population is infected with *Mycobacterium tuberculosis* (MTB), the causative agent of TB. While the majority of these individuals are non-symptomatic and non-contagious carriers of latent TB infection (LTBI), 5–10% will develop symptomatic and highly contagious active disease over their lifetime (Pai et al., 2016; World Health Organization, 2018). Treatment of TB typically requires the use of four antimycobacterial drugs for a minimum duration of 6 months, which is associated with toxicity and/or unpleasant side effects. Treatment of TB has also become increasingly challenging with the rise and spread of multidrug (MDR) and extensively drug-resistant (XDR) TB (Fogel, 2015). Treatment challenges will likely worsen due to the limited arsenal of antimycobacterial drugs available, with only two novel antimycobacterial drugs, bedaquiline and delamanid, being approved by the Food and Drug Administration (FDA) in the past four decades (Tiberi et al., 2019). Alarmingly, spontaneous resistance to bedaquiline and delamanid has already been reported in a number of studies (Bloemberg et al., 2015; Hoffmann et al., 2016). In 2015, the World Health Organization commissioned a global campaign to end TB, which aims to achieve a reduction in deaths and incidence of 95 and 90%, respectively, by 2035 (World Health Organization, 2018). However, despite global efforts, the progress of the End TB strategy has been bottlenecked by insufficient funding (Reid et al., 2019). One of the fundamental pillars of the End TB strategy is to intensify research and innovation, such as the research and development of novel, more effective, and safer antimycobacterial therapeutics. Based on the current trajectory, by 2050, MDR and XDR-TB alone will account for 2.5 million deaths each year (Reid et al., 2019). To minimize a further rise of MDR and XDR-TB and avoid catastrophic numbers of AMR TB mortality, there is an urgent need for the development of new anti-TB agents with novel mechanisms of action.

Animal infection models play a vital role in facilitating the advancement in TB research from pathogenesis to drug development (Zhan et al., 2017). Paradoxically, they are also one of the greatest bottlenecks in TB drug development, with all models being limited by financial sustainability, ethical regulation, research throughput, and physiological capacity to mimic TB disease (Zhan et al., 2017). For example, conventional murine models do not typically develop necrotic granulomas, which are observed in humans. While guinea pigs are able to develop human-like necrotic granulomas, there is a lack in abundance of immunological tools and their larger size increases maintenance costs (Zhan et al., 2017). Furthermore, large numbers of animals are used to screen candidate therapeutic compounds with promising activity *in vitro*, which often do not translate *in vivo* (Tsai et al., 2016). Additionally, some animal models (e.g., mice and non-human primates) can display significant biological variability within and between experiments, which is further influenced by the limited sample size as a consequence of ethical regulations (Fitts, 2011; Zhan et al., 2017). These problems underline the need for a novel, higher throughput, large-scale, drug screening infection model for TB.

We recently determined that the insect *Galleria mellonella* (*G. mellonella*, greater wax moth) can be used as a novel infection model for members of the *Mycobacterium tuberculosis* complex (MTBC) (Li et al., 2018) using *M. bovis* BCG *lux* (BCG *lux*), a vaccine strain incorporating a bioluminescence reporter plasmid (Snewin et al., 1999). BCG *lux* has been extensively validated and characterized under both *in vivo* (mice) and *in vitro* (broth, human whole blood, cell line) conditions and its bioluminescence can be used as a rapid measurement of mycobacterial viability (Kampmann et al., 2000, 2004, 2006; Tena et al., 2003; Martineau et al., 2007a,b,c; Newton et al., 2008, 2011; Andreu et al., 2010; Von Both et al., 2018). BCG *lux* was able to establish a persistent infection over a 2-week time-course with bacteria being internalized by phagocytic hemocytes (functionally similar to neutrophils and macrophages), and the presence of granuloma-like structures. Changes in mycobacterial phenotype within hemocytes were observed with the accumulation of lipid fat bodies, a hallmark of mycobacterial cells in LTBI (Li et al., 2018). Furthermore, *G. mellonella* has been widely used as an infection model to study a large range of pathogens, which include Gram-positive bacteria, e.g., *Streptococcus pyogenes*, *Staphylococcus aureus,* and *Clostridium perfringens* (Desbois and Coote, 2011; Loh et al., 2013; Kay et al., 2019); Gram-negative bacteria, e.g., *Legionella pneumophila*, *Pseudomonas aeruginosa*, and *Escherichia coli* (Harding et al., 2012; Alghoribi et al., 2014; Andrejko et al., 2014); and fungal pathogens, e.g., *Candida albicans* (Sheehan and Kavanagh, 2018). In this report, we demonstrate that *G. mellonella* larvae can also act as a surrogate host to evaluate the efficacy of antimycobacterial therapeutic agents, with the potential to be used as a low-cost, highly reproducible, ethically more acceptable and higher throughput, pre-screening model for drug testing against mycobacteria compared to conventional animal models.

## Materials and Methods

### Mycobacteria, Growth, and Inoculum Preparation

*M. bovis* BCG *lux* (Montréal vaccine strain), transformed with the plasmid pSMT1 containing the *luxAB* genes of *Vibrio harveyi,* was kindly donated by Professor Young’s lab (Snewin et al., 1999). Mid-log phase cultures of BCG *lux* were prepared in Middlebrook 7H9 broth (Difco, US), supplemented with 0.2% glycerol (Sigma-Aldrich, UK), 0.05% polysorbate 80 (Sigma-Aldrich, UK) and 10% albumin dextrose catalase (ADC) as described previously (Li et al., 2018). The bioluminescence of a BCG *lux* culture was measured by a luminometer (Berthold Technologies, DE) using decanal (Sigma-Aldrich, UK) as the substrate (1% v/v in absolute ethanol). Bioluminescence was quantified as relative light units (RLU/ml), which was previously correlated to colony forming units (CFU/ml) at a ratio of 3:1, and is used to rapidly quantify bacterial abundance. BCG *lux* inocula were prepared at 1 × 10^8^ or 1 × 10^9^ CFU/ml in PBS-Tween 80 (PBS-T, 0.05%) (Li et al., 2018).

### Preparation of Antibiotics

Antibiotics were purchased from Sigma-Aldrich. Concentrations were prepared according to the recommended adult human dosages, at a relative body/mass concentration for the larvae (200 mg). First-line drugs: isoniazid (5 mg/kg, INH), rifampicin (10 mg/kg, RIF), pyrazinamide (25 mg/kg, PZA), and ethambutol (15 mg/kg, ETH); and the second-line drug moxifloxacin (6.7 mg/kg, MOX) were used. Relative to mammalian models, these treatment doses are equal to/or lower than what is typically used in mice (Mourik et al., 2017; Lee et al., 2018).

### *G. mellonella* Acquisition, Infection, and Drug Treatment

*G. mellonella* larvae were purchased from Livefoods Direct Ltd. (Sheffield, UK). Healthy larvae were defined as those possessing a uniform cream color that lack discoloration (melanization), high motility with the ability to right themselves when turned over, 250 mg in weight, and 2–3 cm in length (Li et al., 2018). Larval surfaces were decontaminated using 70% ethanol and 10 μl of BCG *lux* was injected into the hemocoel via the last left proleg using a micro syringe (SGE Analytical Sciences, AU). Infected larvae were counted into 25-cm petri dishes lined with filter paper, and incubated in the dark at 37°C. For treatment, a 10 μl dose of a single drug type or drug combination was injected directly into the hemocoel via the last left proleg 1 h post-infection. Where multiple dosing was undertaken, an additional 10 μl dose was injected 24 h post-infection.

### Survival Assay

Survival of infected larvae (*n* = 20 per group) following treatment was recorded every 24 h for 96 h. Larvae were considered dead when they failed to respond to touch. The control groups were infected larvae treated with 10 μl of PBS-T. Kaplan-Meier survival curves were plotted using data pooled from a minimum of two independent experiments.

### Measurement of *in vivo* BCG *lux* Bioluminescence

Larvae (*n* = 30 per group) were infected. At each time point (0–96 h), four larvae were randomly selected and individually homogenized in PBS-T with six 1/8-inch metal beads in a 2 ml lysing tube (MP Biomedical, USA). The RLU of the homogenate was measured using a luminometer to quantify the internal mycobacterial bioluminescence. *In vivo* drug efficacy was determined using two or typically three independent experiments. *In vivo* RLU:CFU was determined to be 4:1 (Li et al., 2018). Homogenate of uninfected larvae was measured to determine background bioluminescence, which was approximately 5,000 RLU/ml.

### Total Hemocyte Count

Larvae (*n* = 20 per group) were injected with 10 μl of PBS-T, INH, or RIF. At 2, 4, and 24 h post-injection, five larvae from each group were bled through needle puncture of the posterior abdomen (approximately 3 drops/60 μl per larva) using a 30 Gauge needle and their hemolymph was pooled into an ice cold Eppendorf to prevent coagulation. Bleeding is a terminal procedure. A volume of 200 μl of the pooled hemolymph was diluted in to PBS containing citrate buffer and 0.37% mercaptoethanol to further prevent coagulation and melanization. Hemolymph mixtures were centrifuged at 1,000 g for 10 min and the cell pellet resuspended in PBS. Then, 10 μl of the cell suspension was further diluted 1:1 with 0.4% trypan blue solution and total hemocyte count (THC) was determined using a hemocytometer. THCs were determined using three independent experiments.

### Minimum Inhibitory Concentration Assay

The *in vitro* minimal inhibitory concentration (MIC), which is defined as the minimal concentration of antimycobacterial drug required to inhibit the growth of BCG *lux*, was determined for this study. A volume of 100 μl of antimycobacterial drugs, prepared in Middlebrook 7H9 broth, was seeded in triplicate into a 96-well plate. Additional 100 μl aliquots of mid-log BCG *lux* at a dilution of 1 × 10^6^ CFU/ml were seeded into each well of the 96-well plate. The positive control consisted of bacterial culture alone; and the negative control consisted of broth alone. The plates were incubated at 37°C in a rocking incubator at 20 rpm for 1 week. Following incubation, optical density of each well was measured in a spectrophotometric plate reader (Molecular Device, UK) at 520 nm. MIC was determined through two independent experiments.

### Statistical Analysis

The datasets were tested for normality using D’Agostino-Pearson omnibus. Data were then analyzed using the non-parametric Kruskal-Wallis test, followed by the Dunn’s multiple comparison test, or Mann-Whitney test. All statistical analyses were carried out using Prism 8 (Graphpad Software Inc., UK).

## Results

**Figure 1:**
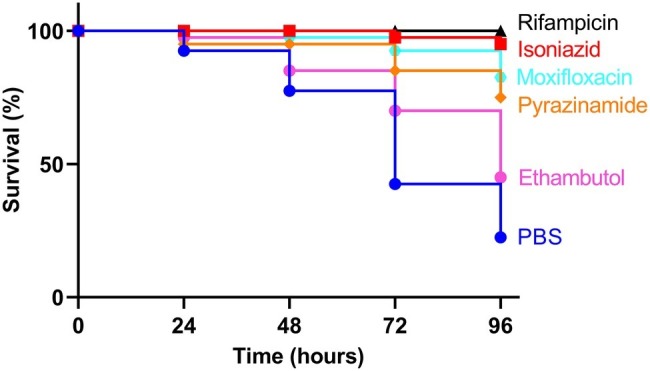
Kaplan-Meier survival curves of *Galleria mellonella* larvae infected with *Mycobacterium bovis* BCG *lux* and treated with first- or second-line antimycobacterial drugs. Larvae (*n* = 20, per group) were infected with 1 × 10^7^ CFU of *M. bovis* BCG *lux*. Following infection, larvae were treated with a single 10 μl dose of isoniazid (5 mg/kg), rifampicin (10 mg/kg), pyrazinamide (25 mg/kg), ethambutol (15 mg/kg), or moxifloxacin (6.7 mg/kg). Control groups consisted of infected larvae, treated with a 10 μl dose of PBS-Tween 80 (0.05%). Survival was monitored every 24 h over a period of 96 h. Data are pooled from a minimum of two independent experiments.

All infected larvae in the control groups showed physiological changes including melanization, reduction in mobility and activity, and observational wasting. Treatment of larvae infected with a lethal dose of BCG *lux,* previously defined as 1 × 10^7^ CFU (Li et al., 2018), using first- or second-line antimycobacterial drugs, resulted in improved survival outcome at all time points over the 96 h time-course with all drugs tested, when compared to the untreated control group (Figure 1). Larvae treated with RIF and INH had the greatest overall survival (100 and 95%, respectively) after 96 h of incubation. By similarly treating healthy naïve larvae with antibiotics alone, we demonstrated that the drug dosages did not result in cytotoxic activity leading to larval mortality (Supplementary Figure S1).

**Figure 2:**
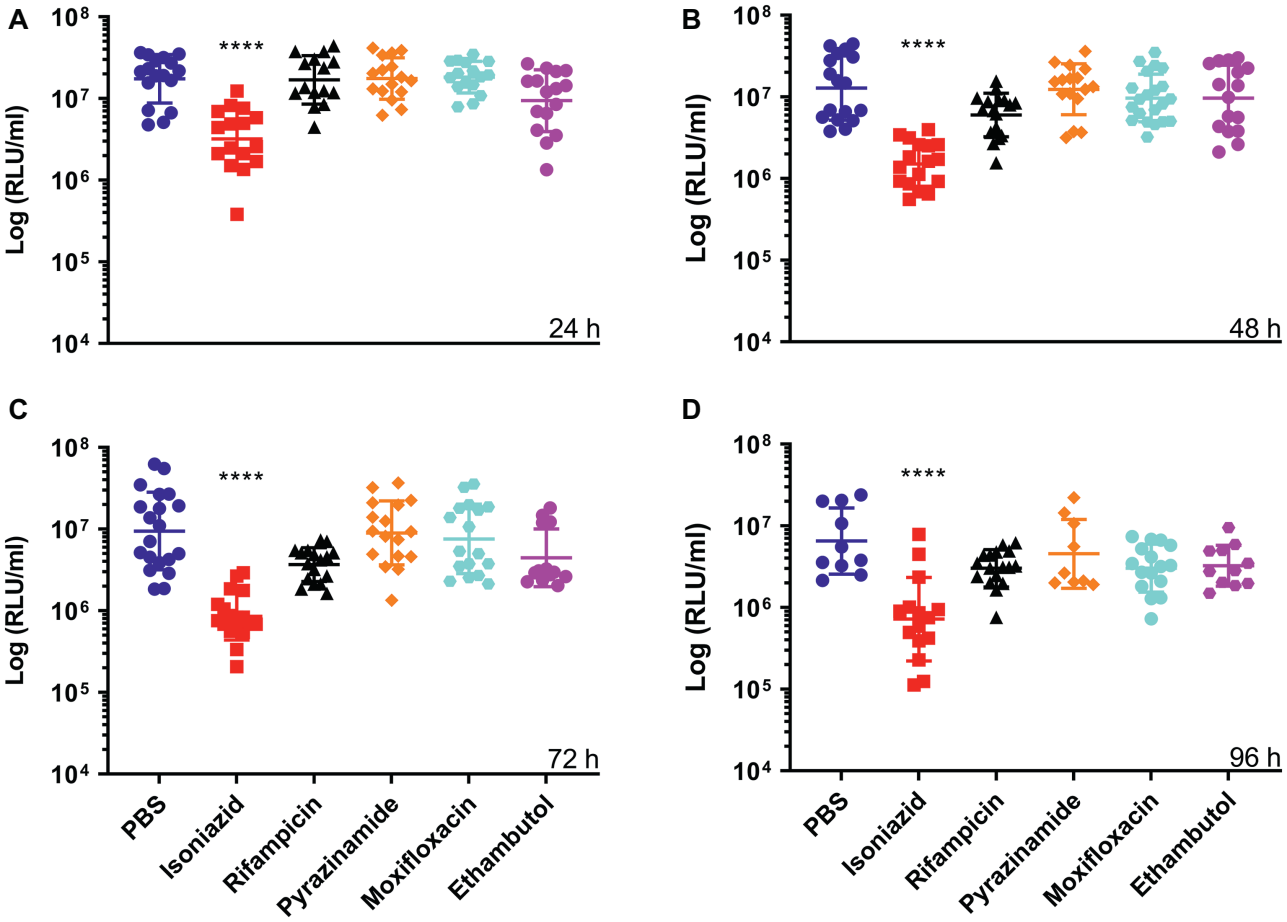
The effect of antimycobacterial drug treatment on the reduction of *Mycobacterium bovis* BCG *lux* bioluminescence within *Galleria mellonella* larvae over 96 h. Larvae (*n* = 30 per group) were each infected with 1 × 10^7^ CFU of *M. bovis* BCG *lux*. At 1 h post-infection, larvae were treated with a single 10 μl dose of isoniazid (5 mg/kg), rifampicin (10 mg/kg), pyrazinamide (25 mg/kg), ethambutol (15 mg/kg), and moxifloxacin (6.7 mg/kg). Control groups consisted of infected larvae similarly treated with a 10 μl dose of PBS-Tween 80 (0.05%). From each group, four larvae were homogenized at **(A)** 24 h, **(B)** 48 h, **(C)** 72 h, and **(D)** 96 h post-infection; and BCG *lux* luminescence of the homogenate was measured using a luminometer and expressed as relative light units (RLU/ml). Data are pooled from a minimum of three independent experiments. Error bars represent the standard deviation of the geometric mean. Kruskal-Wallis test with Dunn’s multiple comparison was carried out against the control. ****p < 0.0001.

**Figure 3:**
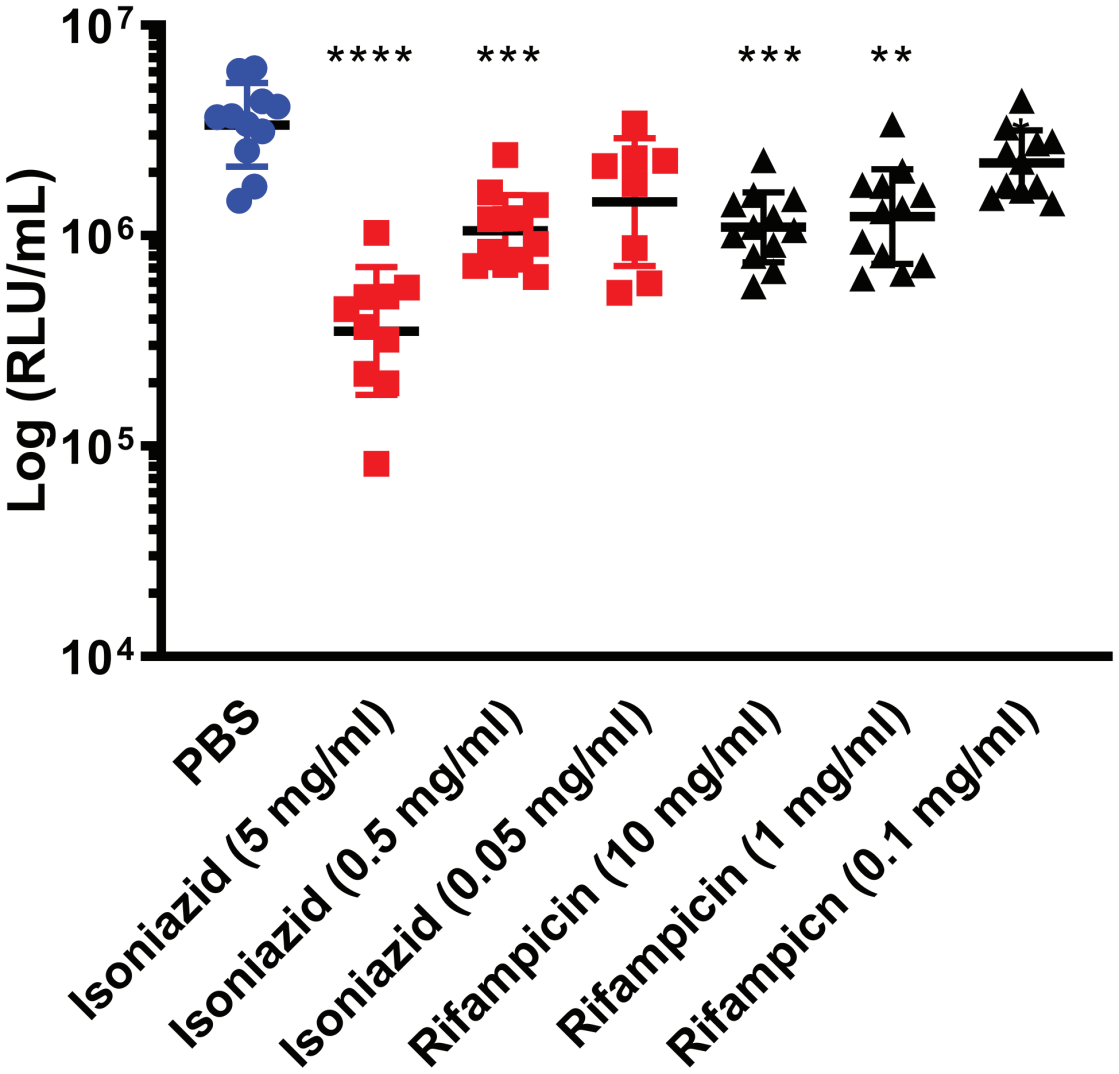
The effect of isoniazid or rifampicin concentration on the reduction of *Mycobacterium bovis* BCG *lux* bioluminescence within *Galleria mellonella*. Larvae (*n* = 30 per group) were each infected with 1 × 10^7^ CFU of *M. bovis* BCG *lux*. At 1 h post-infection, larvae were treated with a single 10 μl dose of isoniazid (5, 0.5, and 0.05 mg/kg) or rifampicin (10, 1, and 0.01 mg/kg). Control groups consisted of infected larvae similarly treated with a 10 μl dose of PBS-Tween 80 (0.05%). From each group, four larvae were homogenized 96 h post-infection and BCG *lux* luminescence of the homogenate was measured using a luminometer and expressed as relative light units (RLU/ml). Data are pooled from a minimum of three independent experiments. Error bars represent the standard deviation of the geometric mean. Kruskal-Wallis test with Dunn’s multiple comparison was carried out against the PBS control. **p < 0.01, ***p < 0.001, ****p < 0.0001.

Furthermore, we demonstrated that an increase or decrease in BCG *lux* bioluminescence in larval lysates can be used to rapidly determine antimycobacterial drug efficacy, when compared to bioluminescence of non-treated infected larvae. In most treatment groups (INH, RIF, ETH, and MOX), we measured a substantial reduction in mycobacterial bioluminescence over the 96 h (Figure 2). INH showed the greatest reduction in mycobacterial bioluminescence, and hence inhibitory activity, at each time point between 24 and 96 h (*p* < 0.0001), with approximately a 1-log or 94% reduction at 96 h compared to the control (Figure 2). Comparison of the mean RIF, ETH, and MOX bioluminescence against the PBS control at 96 h revealed a reduction in mycobacterial bioluminescence of approximately 61%. PZA was the least effective drug with no more than 26% reduction in mean bioluminescence when compared to the control at any given time point. Despite substantial changes in mycobacterial bioluminescence for most drugs, only INH showed a significant reduction in bioluminescence, with RIF, ETH, and MOX showing only a trend in bioluminescence reduction (Figure 2). The use of three varying concentrations of INH (5, 0.5, and 0.05 mg/ml), or RIF (10, 0.1, and 0.01 mg/ml), demonstrated that the drug efficacy observed was dose-dependent (Figure 3). *In vitro* MICs of antimycobacterial drug were determined as follows: INH (0.24 μg/ml), RIF (0.004 μg/ml), PZA (>31.3 μg/ml), ETH (0.98 μg/ml), and MOX (0.015 μg/ml).

**Figure 4:**
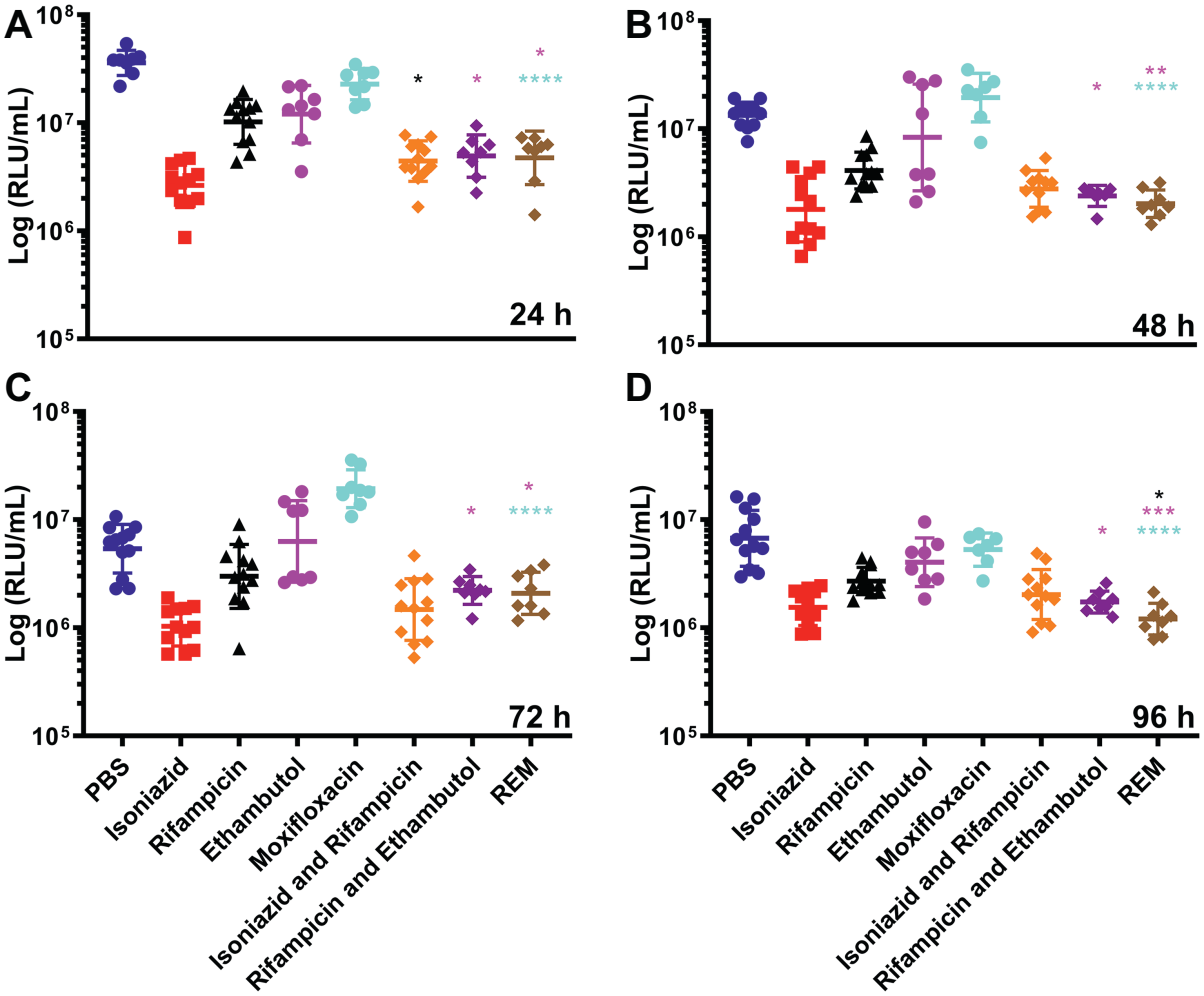
The effect of combined dosing of antimycobacterial drugs on the reduction of *Mycobacterium bovis* BCG *lux* bioluminescence within *Galleria mellonella* larvae over 96 h. Larvae (*n* = 30 per group) were each infected with 1 × 10^7^ CFU of *M. bovis* BCG *lux*. At 1 h post-infection, larvae were treated with a single 10 μl dose of isoniazid (5 mg/kg), rifampicin (10 mg/kg), isoniazid and rifampicin (5 and 10 mg/kg, respectively), rifampicin and ethambutol (10 and 15 mg/kg, respectively) and rifampicin, ethambutol and moxifloxacin (REM) (10, 15, and 6.7 mg/kg, respectively). Control groups consisted of infected larvae similarly treated with a 10 μl dose of PBS-Tween 80 (0.05%). From each group, four larvae were homogenized at **(A)** 24 h, **(B)** 48 h, **(C)** 72 h, and **(D)** 96 h post-infection; and *M. bovis* BCG *lux* luminescence of the homogenate was measured using a luminometer and expressed as relative light units (RLU/ml). Data are pooled from a minimum of two independent experiments. Error bars represent the standard deviation of the geometric mean. Kruskal-Wallis test with Dunn’s multiple comparison was carried out between the combination drug and mono drugs therapy groups. ^*^p < 0.05, ^**^p < 0.01, ^***^p < 0.001, ^****^p < 0.0001, asterisk color correlates to treatment group.

**Figure 5:**
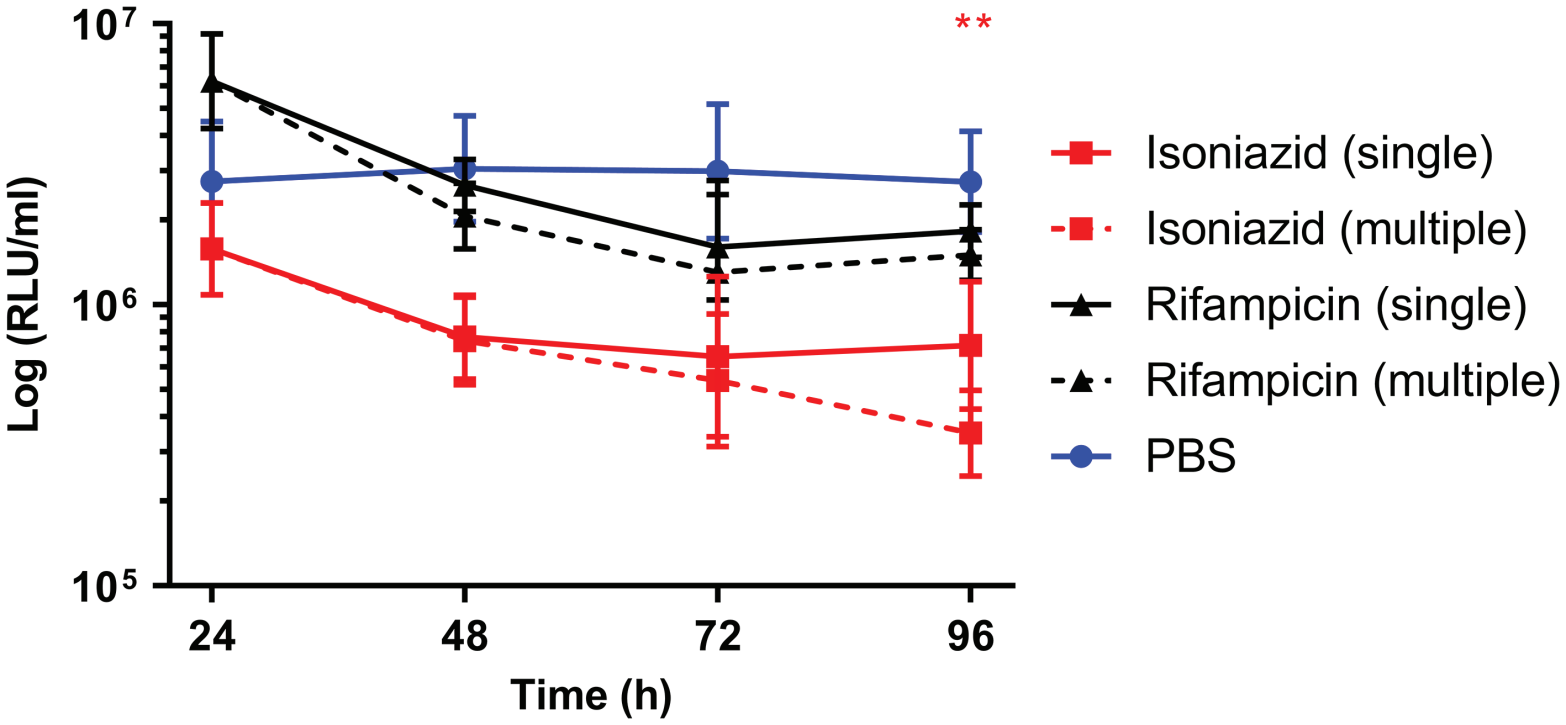
The effect of multiple dosing of isoniazid and rifampicin on the reduction of *Mycobacterium bovis* BCG *lux* bioluminescence within *Galleria mellonella* larvae over 96 h. Larvae (*n* = 30 per group) were each infected with 1 × 10^7^ CFU of *M. bovis* BCG *lux*. For multiple dosing, isoniazid (5 mg/kg) or rifampicin (10 mg/kg) was administered at both 1 h post-infection and 24 h post-infection. Control groups consisted of infected larvae similarly treated with a 10 μl dose/doses of PBS-Tween 80 (0.05%). From each group, four larvae were homogenized at 24, 48, 72, and 96 h post-infection. *M. bovis* BCG *lux* luminescence of the homogenate was measured using a luminometer and expressed as relative light units (RLU/ml). Data are pooled from a minimum of two independent experiments. Error bars represent the standard deviation of the geometric mean. Mann-Whitney test was carried out between single and multiple dosing of each drug type. **p < 0.01, asterisk color correlates to treatment group.

Synergism of dual (INH/RIF and RIF/ETH) and triple (RIF, ETH, and MOX) drug combination therapy was similarly investigated (Figure 4). Use of INH/RIF resulted in a significant reduction in BCG *lux* bioluminescence at 24 h over treatment with RIF alone (*p* < 0.05). Throughout the 96 h time-course, INH/RIF did not improve drug efficacy over the standalone use of INH. RIF in dual (RIF/ETH and RIF/MOX) combination did not yield improved performance over the use of RIF alone. However, triple (RIF/ETH/MOX) combination led to significant improvement in drug efficacy at 96 h (*p* < 0.05) over the standalone use of RIF. Furthermore, in triple combination, both ETH and MOX showed significant improvements in drug efficacy by 96 h (Figure 4). We then determined whether multiple doses of either INH or RIF further reduced BCG *lux* bioluminescence in infected larvae over the course of the 96 h treatment period (Figure 5). There was no significant reduction in bioluminescence with multiple compared to single dosing of RIF over 96 h. For INH, multiple dosing showed little to no difference between 24 and 48 h in BCG *lux* bioluminescence compared to single dosing; however, between 48 and 96 h, there was a trend in improvement and a significant reduction at 96 h (*p* < 0.01).

**Figure 6:**
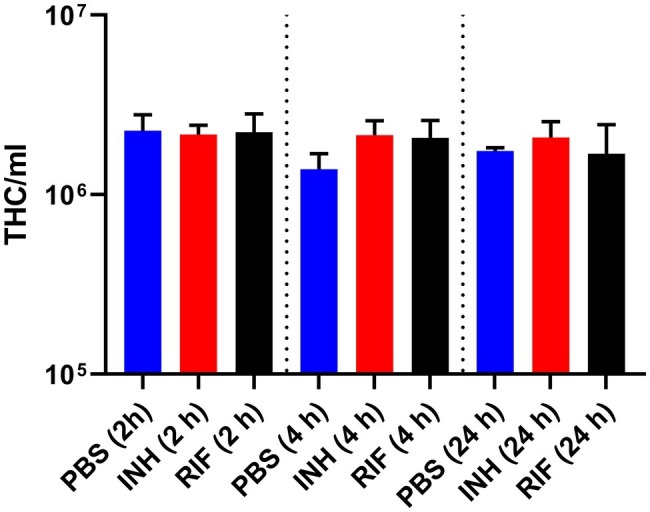
Measurement of total circulating hemocyte density following injection of antimycobacterial drug over 24 h. Naïve healthy *Galleria mellonella* larvae (*n* = 20, per group) were treated with a single 10 μl dose of isoniazid (5 mg/kg) or rifampicin (10 mg/kg). Control groups consisted of larvae, treated with a 10 μl dose of PBS-Tween 80 (0.05%). At 2, 4, and 24 h post-treatment, five larvae from each group were bled, pooled, and the number of total hemocytes (THC) per ml was determined. Data are pooled from a minimum of three independent experiments. Error bars represent the standard deviation of the geometric mean. Kruskal-Wallis test with Dunn’s multiple comparison was carried out against the PBS control.

Previous studies using *G. mellonella* reported activation of innate immunity or immune priming as a result of drug dosing (Entwistle and Coote, 2018; Sheehan and Kavanagh, 2018). Immune priming in our model was investigated by measuring the changes in number of circulating hemocytes in naïve larvae following treatment with INH or RIF. We observed no significant changes in the abundance of circulating hemocytes when compared against the PBS-treated control (Figure 6).

## Discussion

A number of studies have employed *G. mellonella* infection models using bacterial, viral, and fungal pathogens, to determine drug efficacy, dosing, and toxicity (Tsai et al., 2016). *G. mellonella* has thus proven to be a promising *in vivo* infection model, allowing rapid exploratory testing of novel compounds and treatment regimens without ethical constraints, prior to testing in conventional mammalian models (Tsai et al., 2016; Kavanagh and Sheehan, 2018) Furthermore, this model has the capacity to significantly reduce the number of animals used annually in drug pilot studies.

MTB animal research is limited by the prerequisite for containment level (CL) 3 animal facilities, with the associated maintenance costs and ethics (Fonseca et al., 2017). Therefore, to provide a more accessible *in vivo* TB research model, we established the *G. mellonella*-mycobacteria infection model as described previously, with the ability to undertake measurements of changes in host/pathogen interactions, transcriptome, and proteome (Li et al., 2018). Here we have further assessed the potential of *G. mellonella* as a drug screening model by evaluating the efficacy of standard antimycobacterial drugs.

Treatment of BCG *lux* infected larvae led to increased larvae survival and a reduction in BCG *lux* bioluminescence *in vivo*. INH and RIF proved to be the most effective first-line antimycobacterial drugs, which is comparable with studies in mice (Nikonenko et al., 2004; Driver et al., 2012), ETH and MOX showed a trend but non-significant reduction in bioluminescence. The antimycobacterial activity of each drug appeared to be time-dependent, which likely reflects the mode of action and thus the speed of mycobacterial inhibitory activity. In contrast, PZA consistently resulted in the highest bioluminescence of the treated groups and failed to reduce BCG *lux* bioluminescence at any time point, which is likely attributable to its intrinsic resistance to PZA due to deletion of *pncA*. This gene encodes for pyrazinamidase, essential for the conversion of PZA to “active drug,” pyrazinoic acid (POA) (Ritz et al., 2009). While treatment with PZA lead to no more than 26% reduction in *in vivo* bioluminescence at any given point over the 96 h time-course, survival outcome of the PZA-treated group was substantially higher (75% survival) when compared to the PBS-treated control (22.5% survival). Despite the use of PZA as a core drug in the treatment of TB, the precise mechanism and target of the pro-drug remains elusive and still remains a topic of research (Shi et al., 2019). A study by Via and colleagues demonstrated that a substantial quantity of PZA is converted into POA by the host (Via et al., 2015). Recently, PanD (aspartate decarboxylase), involved in Coenzyme A biosynthesis, was identified as a target of POA (Gopal et al., 2017). If PZA is converted into POA by *G. mellonella*, this could account for the low-level efficacy in bioluminescence and improvement in survival outcome. However, PanD inhibition could be circumvented by BCG *lux* as *G. mellonella* are reared using vitamin B_5_ (pantothenate)-rich diet (Finke, 2015), and the presence of exogenous pantothenate has been described to revert this resistance (Gopal et al., 2016). Additionally, POA resistance has also been associated with mutations in phthiocerol dimycocerosate (PDIM) biosynthesis genes *mas* and *ppsA-E* (Gopal et al., 2016), suggesting a potential anti-virulent activity by PZA/POA. Therefore we speculate that host-mediated bioactivation of PZA to POA and/ or the interference with the production of PDIM by PZA/POA may potentially have led to increased survival outcome. This will be a subject for further investigation. The treatment of TB typically requires the use of a combination of antimycobacterial drugs, administered on multiple occasions (World Health Organization, 2010). We therefore investigated multiple drug dosing and combination drug therapy, using the *G. mellonella* model. Both combined, and multiple dosing, showed a trend in improved antimycobacterial activity over monotherapy and single dosing. The lack of a significant reduction in BCG *lux* luminescence with combination drug therapy over the use of single drug was not surprising, as the primary aim of combined drug therapy is to minimize the risk of drug resistance development (World Health Organization, 2010). Further work will determine the usage of *G. mellonella* for screening novel antimycobacterial agents and their synergistic activities with frontline drugs. Additionally, we observed a drug dose-dependent efficacy at three varying doses of INH and RIF, which clearly highlights the sensitivity of this model to changes in drug dosing. Therefore, we suggest that researchers should test various doses of their drugs of interest to determine the optimum dose. There is little information on the pharmacokinetics and pharmacodynamics of antibiotics in *G. mellonella* (Thomas et al., 2013), and no data to our knowledge with antimycobacterial drugs. Further studies are warranted and likely to increase the uptake of the *G. mellonella*-mycobacterial model.

The use of BCG over *Mycobacterium smegmatis* as a surrogate for MTB was rationalized by its genetic similarity. BCG shares >99.99% genetic similarity to MTB, while *M. smegmatis* shares roughly 70% of its genome with MTB (Garnier et al., 2003; Mohan et al., 2015; Yuan and Sampson, 2018). Furthermore, when 2,000 compounds were screened against MTB, BCG, and *M. smegmatis*, approximately 80% of compounds that were active in BCG showed efficacy against MTB, while only 50% of the compounds that were active in *M. smegmatis* displayed efficacy against MTB (Altaf et al., 2010). Therefore, BCG appears to be a more superior surrogate mycobacterial organism for drug screening against MTB when compared to *M. smegmatis*.

Drug efficacies for non-tuberculous mycobacteria (NTM) such as *Mycobacterium marinum*, *Mycobacterium aurum*, *Mycobacterium fortuitum,* and *Mycobacterium abscessus* have been reported in the *G. mellonella* model. However, comparisons between mycobacterial species are challenging as the drug efficacies and susceptibilities of NTM are different to those observed for the MTBC (Entwistle and Coote, 2018; Meir et al., 2018).

The innate immune system of *G. mellonella* is highly sensitive to external factors ranging from thermal, physical, and chemical stress (Browne et al., 2014; Sheehan and Kavanagh, 2018). Browne et al. highlighted that innate immune priming can occur in response to thermal stress, leading to a transient increase in circulating hemocyte density and production of antimicrobial peptides in anticipation of infection. A similar priming response can also occur in response to antibiotic treatment (Entwistle and Coote, 2018; Sheehan and Kavanagh, 2018). However, in our *G. mellonella* model, the use of INH and RIF, the most efficacious drugs, led to non-significant minor or no changes in circulating hemocyte density when compared to the PBS-treated control. Regardless of our observation, the possibility of immune priming should be explored when testing any new drugs in this model, as this may influence the outcome of the experiment. Antimicrobial peptides (AMP) are additional immune factors that should be taken into consideration when using this model for drug screening. Approximately 20 putative AMP have thus far been identified in *G. mellonella* (Pereira et al., 2018). Synergism of *G. mellonella* AMP with antibiotics has not yet been described. However a number of studies have reported synergism between AMP of other invertebrate and antibiotics, e.g., AMP of *Apis mellifera* with vancomycin (Akbari et al., 2019) or AMP of *Aedes aegypti* with tetracycline (Zheng et al., 2017). As such, the production and abundance of host AMP and the potential synergism with conventional antibiotics for treatment of TB will be investigated in the near future as a separate proteomic study.

The establishment of infection prior to treatment in a host organism is an important factor to consider when defining the true efficacy of the drugs tested *in vivo*, and the time required for the establishment of infection can vary between models, e.g., establishment of infection in mouse model will generally take longer than those seen in *Drosophila melanogaster* (fruit fly) or *Danio rerio* (zebrafish) (Dionne et al., 2003; Myllymäki et al., 2016; Zhan et al., 2017). Additionally, the incubation period following infection can be altered to further mimic types of infection, e.g., active TB infection or LTBI in certain models. For this particular study, 1 h post-infection was chosen as a point of treatment for two reasons. Firstly, a previous study has shown that phagocytosis of mycobacteria occurs within 1 h post-infection demonstrated through the Fluorescein isothiocyanate (FITC) labeled non-tuberculous mycobacteria (NTM) phagocytic assay using similar inocula density (3 × 10^7^ CFU) (Entwistle and Coote, 2018). Secondly, the standard procedure for the treatment of *G. mellonella* following infection is typically 30 min to 2 h post-infection (Tsai et al., 2016). Nonetheless, we do acknowledge the potential merits of extended incubation prior to treatment. For example, Meir and colleagues (Meir et al., 2018) started treatment of *M. abscessus* infected *G. mellonella* at 24 and 48 h post-infection. However, this required the use of a low dose of bacterial inocula (1 × 10^3^ CFU) to accommodate the required incubation period. A potential downside of the use of extended incubation time prior to treatment may be the possible accumulation or time-dependent induction of AMP within the hemocoel (Sheehan et al., 2019). In future studies, the effect of treatment timing on drug efficacy will be evaluated.

As briefly mentioned above, other non-mammalian infection models such as *D. melanogaster* and *D. rerio* have similarly been used for antimycobacterial drug screening (Dionne et al., 2003; Oh et al., 2013; Ordas et al., 2015). However, both the fruit fly and zebrafish are biologically incompatible with the MTBC and require the use of *M. marinum* as a surrogate organism for MTB (Myllymäki et al., 2015; Zhan et al., 2017). In contrast, both MTBC and NTM species can be used with *G. mellonella* (Entwistle and Coote, 2018; Meir et al., 2018), providing a broader variety of compatible mycobacterial species for use when screening antimycobacterial compounds. While *M. marinum* and MTBC share significant similarities in the expression of virulence factors and other physiological properties (Tobin and Ramakrishnan, 2008), antimycobacterial drug efficacy against NTM does not always lead to efficacy in MTBC. Furthermore, the ability to study mycobacterial responses under human physiological conditions (e.g., 37°C) during drug treatment using MTBC is highly desirable, as temperature can affect mycobacterial physiology (Flentie et al., 2016). Infection of both *D. melanogaster* and *D. rerio* typically requires the use of a costly microinjector, where bacterial inocula/drugs are delivered through a glass capillary tube under microscopy due to their size. For novice users, this method of infection can be difficult and training and familiarity with the technique become essential for throughput and experimental outcome (Benard et al., 2012; Khalil et al., 2015). For infection of *D. rerio,* recent advances now allow for a non-invasive natural method of infection through bathing (Dalton et al., 2017). In comparison, the BCG *lux*-*G. mellonella* infection model requires minimal training, the prerequisite of infection is an inexpensive micro syringe alone, and the size of *G. mellonella* (2–3 cm) makes handling and manipulation of the larvae during injection effortless (Asai et al., 2019).

## Conclusion

In conclusion, this proof-of-principle study has shown that *G. mellonella* combined with the use of a bioluminescent reporter mycobacterial species allows for the rapid determination of efficacy of antimycobacterial agents and drug regimens. There are a number of advantages over pre-existing non-mammalian infection models, such as relative ease of infection for novice users and the ability to utilize MTBC over NTM. The successful uptake of this model as a drug screen within the TB research community could accelerate drug development while significantly reducing and replacing the use of animal models in TB drug research and development.

## Data Availability Statement

The datasets generated for this study are available on request to the corresponding author.

## Author Contributions

MA, YL, and JK contributed to acquisition, analysis, and interpretation of data for the work. MA wrote the first draft. All authors contributed to the conception and design of the work and to manuscript revision, and read and approved the submitted and final version.

### Conflict of Interest

The authors declare that the research was conducted in the absence of any commercial or financial relationships that could be construed as a potential conflict of interest.
