# Supplementary figures and images for: Galleria mellonella: An Infection Model for Screening Compounds Against the Mycobacterium tuberculosis Complex
Source: Front Microbiol. 2019 Nov 20;10:2630. doi: 10.3389/fmicb.2019.02630 (PMC6882372; doi:10.3389/fmicb.2019.02630)

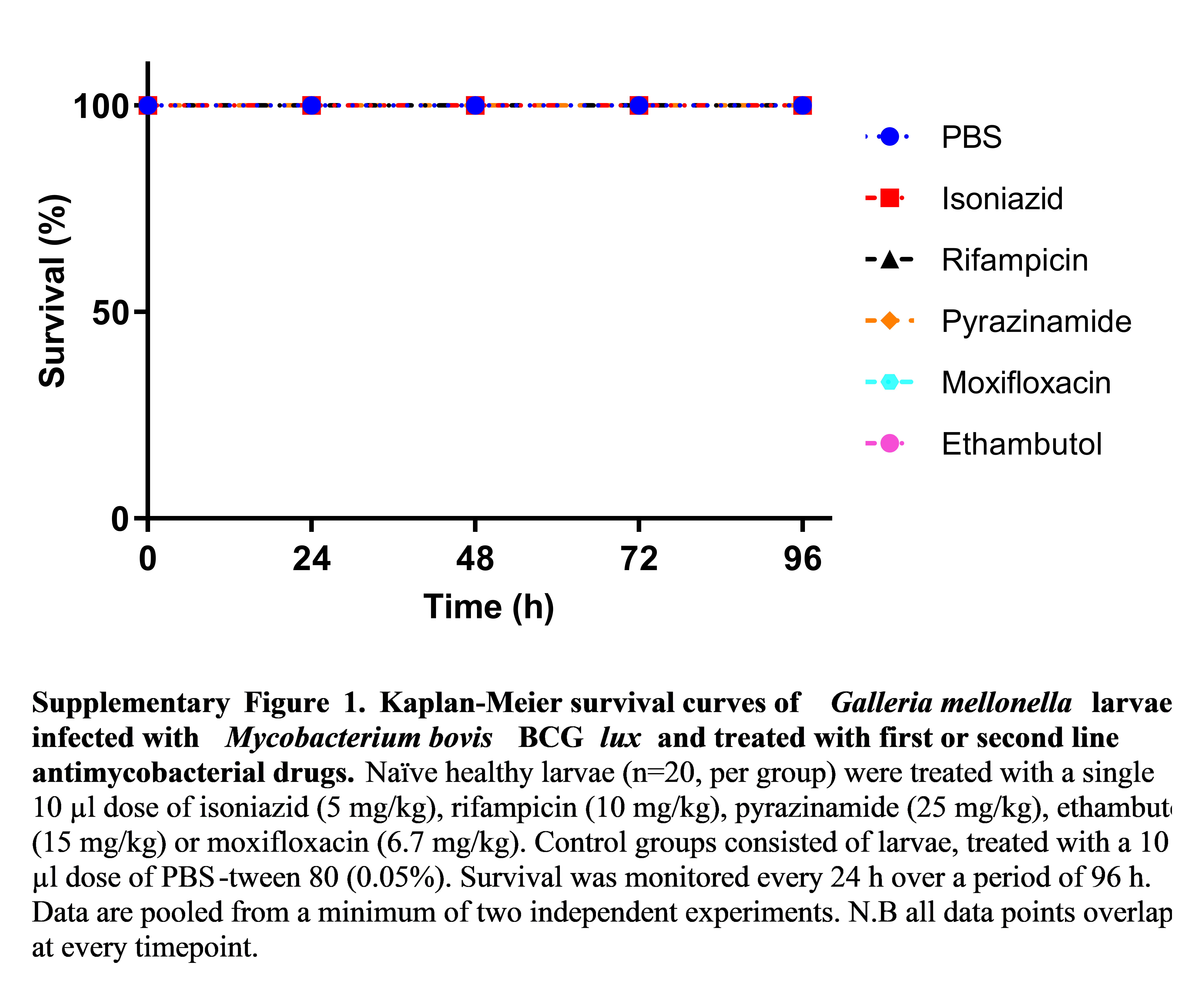

Supplement: Supplementary file 1 [file Image_1.TIF]
